# Acute Kidney Injury in Severe Sepsis and Septic Shock in Patients with and without Diabetes Mellitus: A Multicenter Study

**DOI:** 10.1371/journal.pone.0127411

**Published:** 2015-05-28

**Authors:** Marion Venot, Lise Weis, Christophe Clec’h, Michael Darmon, Bernard Allaouchiche, Dany Goldgran-Tolédano, Maité Garrouste-Orgeas, Christophe Adrie, Jean-François Timsit, Elie Azoulay

**Affiliations:** 1 Service de Réanimation Médicale, AP-HP, Hôpital Saint-Louis, Paris, France; 2 Service de Médecine Vasculaire et Hypertension artérielle, AP-HP, Hôpital Européen Georges Pompidou, Paris, France; 3 Service de Réanimation, AP-HP, Hôpital Avicenne, Paris, France; 4 Faculté de Médecine, Université Paris 13, Bobigny, France; 5 Service de Réanimation Médicale, Centre Hospitalier Universitaire de Saint-Etienne, Saint-Etienne, France; 6 Faculté de Médecine Jacques Lisfranc, Université Jean Monnet, Saint-Etienne, France; 7 Service de Réanimation Chirurgicale, Centre Hospitalier Universitaire Edouard Herriot, Lyon, France; 8 Service de Réanimation Polyvalente, Centre Hospitalier de Gonesse, Gonesse, France; 9 Service de Réanimation Polyvalente, Groupe Hospitalier Paris Saint-Joseph, Paris, France; 10 Service de Physiologie, AP-HP, Hôpital Cochin, Paris, France; 11 Service de Réanimation Polyvalente, Centre Hospitalier Universitaire de Grenoble, Grenoble, France; 12 U 823, Université de Grenoble 1, Grenoble, France; 13 Faculté de Médecine, Université Paris 5, Paris, France; Bambino Gesù Children's Hospital, ITALY

## Abstract

**Introduction:**

Whether diabetes mellitus increases the risk of acute kidney injury (AKI) during sepsis is controversial.

**Materials and Methods:**

We used a case-control design to compare the frequency of AKI, use of renal replacement therapy (RRT), and renal recovery in patients who had severe sepsis or septic shock with or without diabetes. The data were from the Outcomerea prospective multicenter database, in which 12 French ICUs enrolled patients admitted between January 1997 and June 2009.

**Results:**

First, we compared 451 patients with severe sepsis or septic shock and diabetes to 3,277 controls with severe sepsis or septic shock and without diabetes. Then, we compared 318 cases (with diabetes) to 746 matched controls (without diabetes). Diabetic patients did not have a higher frequency of AKI (hazard ratio [HR], 1.18; *P* = 0.05]) or RRT (HR, 1.09; *P* = 0.6). However, at discharge, diabetic patients with severe sepsis or septic shock who experienced acute kidney injury during the ICU stay and were discharged alive more often required RRT (9.5% vs. 4.8%; *P* = 0.02), had higher serum creatinine values (134 vs. 103 µmoL/L; *P*<0.001) and had less often recovered a creatinine level less than 1.25 fold the basal creatinine (41.1% vs. 60.5%; *P*<0.001).

**Conclusions:**

In patients with severe sepsis or septic shock, diabetes is not associated with occurrence of AKI or need for RRT but is an independent risk factor for persistent renal dysfunction in patients who experience AKI during their ICU stay.

## Introduction

Diabetes mellitus affects 10% of the general population [1] and is associated with high morbidity and mortality rates. Sepsis and renal dysfunction are among the main complications of diabetes [2; 3]. Chronic kidney disease (CKD) is the most common renal dysfunction pattern in diabetes and may progress to kidney failure [4]. In industrialized countries, diabetes-related CKD is the leading cause of end-stage kidney failure [4].

The risk of acute kidney injury (AKI) during life-threatening events such as sepsis is increased in patients with diabetes, even those with the milder stages of CKD manifesting as small decreases in the glomerular filtration rate (GFR) [5]. Diabetes has been identified as an independent risk factor for AKI [6, 7]. Moreover, acute-on-chronic kidney injury is associated with a need for long-term dialysis, failure to recover initial renal function, and death [8]. Sepsis is a major cause of AKI, which develops in one-fourth of all patients with sepsis and half of patients with bacteremia or shock [9]. Sepsis-related AKI is associated with high mortality rates of up to 70% [9, 10, 11, 12]. Whether diabetes increases the risk of AKI during sepsis is controversial [13, 14]. However, diabetes is an established risk factor for both AKI and sepsis [2, 3, 4].

Here, our objective was to evaluate the impact of diabetes on kidney function in patients with severe sepsis or septic shock. We used a case-control design to compare the incidence of AKI, use of renal replacement therapy (RRT), and renal recovery in diabetic and nondiabetic patients with severe sepsis or septic shock enrolled in a prospective database by 12 intensive care units (ICUs).

## Materials and Methods

### Study design and data source

Outcomerea is a prospective observational multicenter database established between January 1997 and June 2009 by 12 French ICUs. To avoid selection bias and ensure external validity, a random sample of patients older than 16 years of age and admitted for >24 hours were entered into the database each year. Participating centers could enroll either consecutive patients admitted to a prespecified number of beds throughout each year or all consecutive patients admitted during a prespecified month. The number of beds or month of enrolment were decided yearly by the database steering committee. Detailed clinical and outcome data were recorded prospectively for each patient, on a daily basis, by senior ICU physicians with assistance from trained study monitors in each participating ICU. Data were recorded at baseline (including demographic characteristics, comorbidities, baseline severity, admission diagnosis, admission category, and patient location just before ICU admission) then on each ICU day (including diagnostic and therapeutic procedures, laboratory parameters, organ failures, sepsis, iatrogenic events, and treatment-limitation decisions). For each patient, the data were entered into an electronic case-report form using VIGIREA and RHEA data-capture software (Outcomerea, Rosny-sous-Bois, France) and all case-report forms were then entered into the Outcomerea data warehouse. The data-capture software automatically conducted multiple checks for internal consistency of most of the variables at entry into the database. Queries generated by these checks were resolved with the source ICU before incorporation of the new data into the database. At each participating ICU, data quality was controlled by having a senior physician from another participating ICU check a 2% random sample of the study data. A 1-day coding course was held annually with the study investigators and study monitors.

In accordance with French law, approval for the development and maintenance of the Outcomerea database was obtained from the data confidentiality agency Commission Nationale de l’Informatique et des Libertés. The study was approved by the ethics committee in Clermont-Ferrand, France. Given the observational study design, absence of effect of data collection on patient management, and use of anonymized data for the statistical analyses, the ethics committee waived the requirement for informed consent from study patients. The senior physicians and centers that participated in Outcomerea are listed in the acknowledgment section.

### Study population

Cases were consecutive patients with severe sepsis or septic shock and diabetes. Controls were consecutive patients with severe sepsis or septic shock and no diabetes. Diabetic status was assessed based on previous medical history as reported by patients, relatives or consultants. We excluded patients with CKD (MDRD eGFR less than 60ml/min, when baseline creatinine was available), prerenal AKI (rapidly reversible AKI less than 48 hours), extrarenal indications for dialysis (tumor lysis syndrome, Metformine related lactic acidosis), or treatment-limitation decisions. We compared all cases to all controls then a subset of cases to matched controls.

### Matching procedure

Using an algorithm (available at: http://www.outcomerea.org/Macros-SAS/Voir-categorie.html), diabetic patients (exposed population) were matched to nondiabetic patients (unexposed population) on the following risk factors for CKD and AKI: age (±5 years), SAPS II score (±5 points), and underlying cardiac comorbidity (New York Heart Association (NYHA) class IV heart failure) assessed according to Acute Physiology and Chronic Health Evaluation (APACHE) II definitions. Additional matching criteria were center and period of admission (1997–2002 and 2003–2009) to account for variations in RRT practices over time and across centers. Up to 3 controls could be selected for each case.

### Endpoints and analysis

Comparisons between cases and controls focused on three points: occurrence of AKI defined as any KDIGO category (using the only serum creatinine criteria), need for RRT, and renal function at discharge as assessed by the KDIGO category, creatinine level, weaning from RRT, and renal recovery defined as a creatinine level less than 1.25 fold the basal creatinine.

### Statistical analysis

Results are described as number (%) for categorical variables and mean±SD or median [interquartile range] for continuous variables. Comparisons of all cases and controls relied on chi-square tests for categorical data and on Student’s t-test or Wilcoxon’s test for continuous data, as appropriate. Comparisons in the matched case-control analysis involved univariate conditional logistic regression followed by multivariate conditional logistic regression to assess associations between diabetes and both AKI and need for RRT, adjusting for potential confounding variables (namely, common risk factors for AKI such as hemodynamic failure or nephrotoxic drugs (Vancomycin, aminoglycosides and contrast agents). Wald χ2 tests were used to determine the significance of each variable. Adjusted odds ratios (ORs) and 95% confidence intervals (CIs) were calculated for each parameter estimate.

*P* values less than 0.05 were considered significant. Analyses were performed using the SAS 9.1 software package (SAS Institute, Cary, NC, USA).

## Results

**Fig 1:**
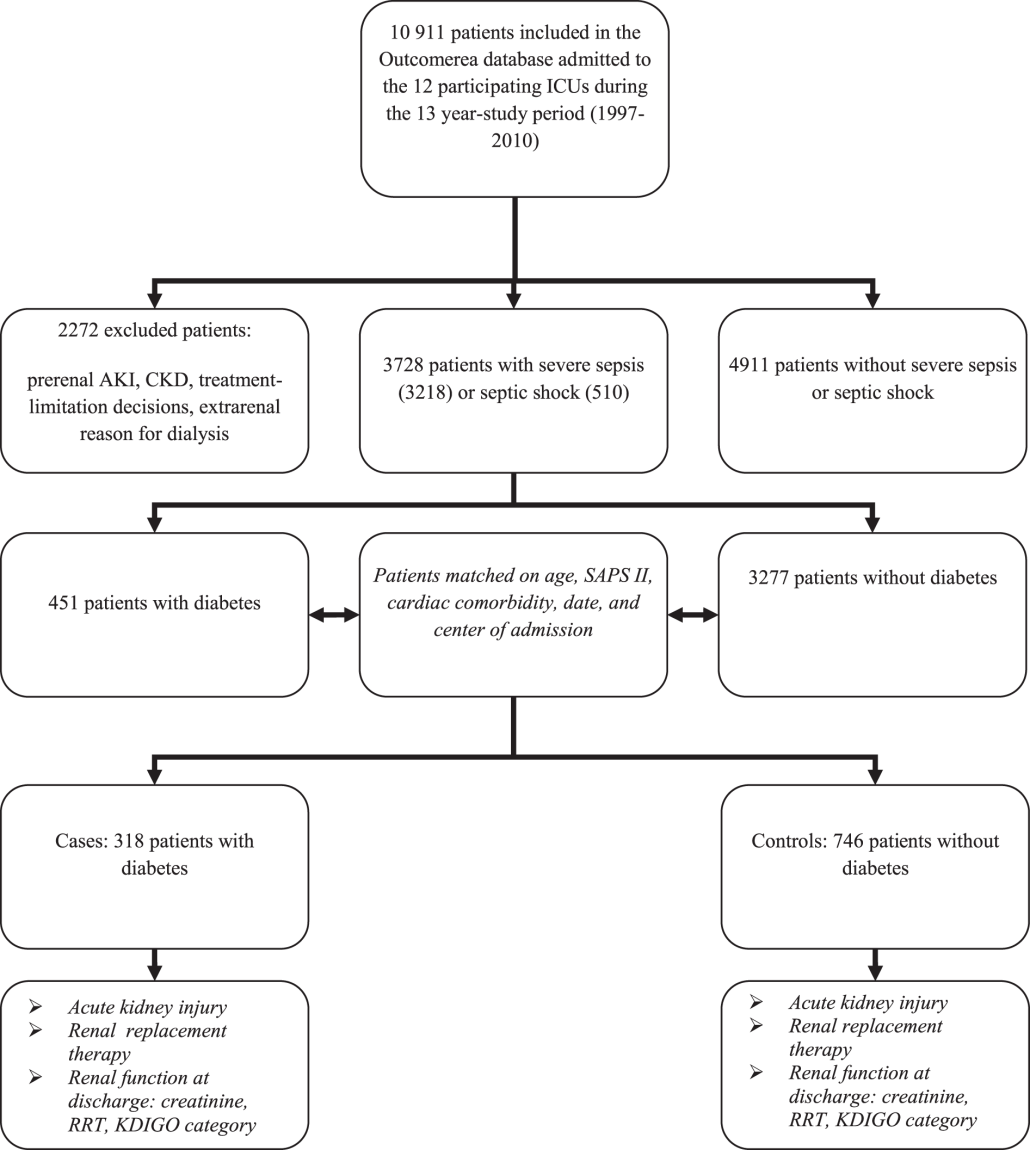
Patient flow chart.

As shown in Fig 1, among the 10 911 patients admitted to the 12 participating ICUs over the 13-year study period (1997–2010), 3728 had severe sepsis (n = 3218) or septic shock (n = 510). Among them, 451 had diabetes and 3277 did not. For the matched analysis, 318 (71%) cases had 746 matched controls.

### Patient characteristics

Table 1 reports the baseline characteristics of the cases and controls. The acute illness was more severe and cardiac comorbidities more common in the group with diabetes. Mortality was higher in the diabetic patients but the difference was not statistically significant (19.8% vs. 15% in the matched case-control analysis; *P* = 0.08).

**Table 1:** Baseline characteristics of patients with severe sepsis or septic shock with (cases) or without (controls) diabetes mellitus.

	All cases and controls			Matched cases and controls		
	Diabetes (N = 451)	No diabetes (N = 3277)	*P* value	Diabetes (N = 318)	No diabetes (N = 746)	*P* value
**Age, mean (SD)**	67.3 (12.2)	61.7 (16.5)	< 0.001	67.6 (11.7)	67.3 (12.5)	0.7
**Males, n (%)**	249 (61.9)	2062 (62.9)	0.7	197 (62.8)	490 (65.7)	0.2
**SAPS II score, mean (SD)**	53.7 (21.2)	48.1 (19.7)	< 0.001	50.6 (17.10)	48.6 (16)	0.06
**APACHE II score, mean (SD)**	20.6 (7.3)	18.7 (7.2)	< 0.001	20.1 (6.8)	18.9 (6.5)	0.02
**Transfer from ward, n (%)**	223 (49.5)	1649 (50.3)	0.7	156 (49.1)	401 (53.8)	0.2
**McCabe, n (%)**			0.5			0.1
**1**	261 (58)	1852 (56.5)		173 (54.4)	445 (59.6)	
**2**	149 (33)	1206 (36.8)		126 (39.6)	266 (35.7)	
**3**	41 (9)	219 (6.7)		19 (6)	35 (4.7)	
**Admission category, n (%)**			**0.007**			0.1
**Medical**	341 (75.6)	2238 (68.3)		233 (73.3)	492 (66.1)	
**Scheduled surgery**	68 (15.1)	629 (19.2)		54 (17)	160 (21.4)	
**Unscheduled surgery**	42 (9.3)	410 (12.5)		31 (9.7)	94 (12.5)	
**Chronic comorbidities, n (%)**						
**Heart disease**	91 (20.2)	392 (12)	< 0.001	65 (20.4)	75 (10)	< 0.001
**Respiratory disease**	69 (15.3)	457 (14)	0.4	45 (14.1)	116 (15.6)	0.7
**Liver disease**	45 (10)	238 (7.3)	0.04	34 (10.7)	52 (7)	0.07
**Immunodeficiency**	53 (11.8)	571 (17.4)	0.002	40 (12.6)	112 (15)	0.3
**ICU mortality, n (%)**	99 (22)	674 (20.6)	0.5	63 (19.8)	112 (15)	0.08
**ICU stay length, days (SD)**	11.6 (12.8)	13.2 (15.2)	0.02	9.1 (8.5)	16.8 (17.5)	0.01

### Occurrence of acute kidney injury (AKI) and use of renal replacement therapy (RRT)

Both AKI and use of RRT were significantly more common in the patients with diabetes in the analysis comparing all cases to all controls (Table 2).

**Table 2:** Acute kidney injury and renal replacement therapy in patients with severe sepsis or septic shock: comparison of all cases (with diabetes) and controls (without diabetes).

	Diabetes (N = 451)	No diabetes (N = 3277)	*P* value
**Acute kidney injury, n (%)**	327 (72.5)	1832 (55.9)	<0.001
**Renal replacement therapy, n (%)**	93 (20.6)	452 (13.8)	<0.01

However, no difference in the frequency of AKI was found in the matched case-control analysis (Table 3), even after adjustment for nephrotoxic drugs, duration of hemodynamic failure, and chronic comorbidities. When we confined the matched analysis to cases with insulin-dependent diabetes and their controls, we found a significantly higher frequency of AKI in the diabetic patients, but this difference was no longer apparent after adjustment for nephrotoxic drugs, duration of hemodynamic failure, and underlying condition. Similarly, the use of RRT was not more common in the cases in the matched case-control analysis (Table 3), even after adjustment for nephrotoxic drugs, duration of hemodynamic failure, and chronic comorbidities. Confining this analysis to cases with insulin-dependent diabetes and their controls did not show a difference in the use of RRT.

**Table 3:** Odds ratios for acute kidney injury and renal replacement therapy associated with diabetes mellitus in the matched case-control analysis of patients with severe sepsis or septic shock: unadjusted and adjusted conditional logistic regression models.

	Matched patients		After adjustment	
	OR [95%CI]	*P* value	OR [95%CI]	*P* value
**Acute kidney injury**				
All patients with diabetes	1.18 [1–1,38]	0.05	1.24 [0.79–1.94]	0.34
Insulin-dependent diabetes	1.37 [1.04–1.81]	0.02	2.51 [0.94–6.65]	0.07
Non-insulin-dependent diabetes	1.09 [0.89–1.33]	0.4	1.05 [0.64–1.72]	0.9
**Renal replacement therapy**				
All patients with diabetes	1.09 [0.79–1.49]	0.6	1.03 [0.51–2.08]	0.9
Insulin-dependent diabetes	1.09 [0.62–1.91]	0.8	2.08 [0.42–10.12]	0.4
Non-insulin-dependent diabetes	1.09 [0.74–1.59]	0.7	0.87 [0.40–1.92]	0.7

OR, odds ratio; 95% CI, 95% confidence interval

### Renal function at discharge (Table 4)

In the overall cohort, 244 (74.6%) of 327 patients with diabetes and AKI were discharged alive compared to 1312 (71.6%) of 1832 patients with AKI and without diabetes. Neither use of RRT nor renal function recovery were significantly different between all cases and all controls. Serum creatinine at discharge in patients who did not need RRT was significantly higher in diabetic than nondiabetic patients. Proportions of patients with KDIGO category 3 at discharge were not different between the two groups.

**Table 4:** Renal function at ICU discharge in patients with severe sepsis or septic shock who experienced acute kidney injury during the ICU stay and were discharged alive.

	All cases and controls			Matched cases and controls		
	Diabetes (N = 244)	No diabetes (N = 1312)	*P* value	Diabetes (N = 174)	No diabetes (N = 357)	*P* value
**Serum creatinine (μmol/L), median, [interquartile range]**	127 [93–206]	115 [79–184]	< 0.01	134 [104–239]	103 [75–155]	< 0.001
**Recovery of renal function, n (%)**	114 (48.1)	700 (54.4)	0.08	69 (41.1)	214 (60.5)	< 0.001
**Persistent renal dysfunction at ICU discharge**						
**KDIGO category 3, n (%)**	54 (22.8)	225 (17.5)	0.05	43 (25.7)	53 (15)	< 0.01
**Patients still on RRT, n (%)**	20 (8.9)	72 (5.6)	0.05	16 (9.5)	17 (4.8)	0.02

RRT, renal replacement therapy

Comparison of all cases (with diabetes) and controls (without diabetes) and comparison of matched cases and controls

In the matched population, 174 of 225 (77.3%) patients with diabetes and AKI were discharged alive compared to 357 of 446 (80%) patients with AKI and without diabetes. Compared to controls, the cases had a higher proportion of patients requiring RRT at discharge, a smaller proportion having recovered their previous renal function, higher serum creatinine levels, and a higher proportion of patients with KDIGO category 3.

## Discussion

Diabetes mellitus is a highly prevalent condition [1] associated with both AKI and sepsis [2, 3, 4]. Few studies assessed the impact of having diabetes on the risk of AKI during sepsis, which is a current focus of controversy [13, 14]. We conducted a multicenter study of prospectively collected data using a matched case-control design to determine whether diabetes increased the risk and worsened the prognosis of AKI during severe sepsis or septic shock. Our results showed that having diabetes did not increase the risk of AKI or RRT but worsened the renal prognosis at discharge, as assessed based on need for RRT, serum creatinine level, and recovery of previous renal function.

Among patients admitted to the ICU, 35% to 70% develop AKI, which is related to sepsis in half the cases. AKI has been reported in 19% of patients with sepsis, 23% of those with severe sepsis, and 51% of those with septic shock and bacteremia [4, 9]. In a prospective multicenter study, 70% of patients with AKI and sepsis died, compared to 45% of those with AKI and no sepsis [10]. The development of AKI during sepsis has been reported to increase mortality (OR, 1.53) and hospital stay length [11, 12]. Given this major impact of AKI during sepsis, determining the effect of diabetes on the risk and prognosis of AKI is of considerable interest. Diabetes is an established independent risk factor for AKI [6, 7], both overall and during sepsis [14], and the risk of sepsis of any type is 2.5-fold to 6-fold higher in patients with diabetes than in the general population [3, 15, 16, 17, 18, 19]. Diabetes increased the risk of death from infectious causes in one study [20] but did not affect mortality in a study focusing on severe sepsis [21]. Pre-existing kidney diseases such as diabetes-related CKD may increase the risk of developing AKI during sepsis [22] [23], and, diabetes itself may also increase the risk of AKI during sepsis. In a prospective single-center ICU study, elevated serum creatinine and liver failure on the first day of severe sepsis were associated with subsequent AKI, whereas diabetes was not [13].

In our study, diabetes decreased the likelihood of renal function recovery in patients with severe sepsis or septic shock. Clinical practical consequences of this information might be to consider involvement of a nephrologist in the early ICU care of patients with diabetes and severe sepsis or septic shock as well as long-term follow-up by a nephrologist after ICU discharge to determine whether these patients eventually recover their previous level of renal function. Diabetic patients admitted for sepsis severe or septic shock should have explorations including urinary sediment and nephrotoxic drugs should be used with caution.

Our study has several limitations however. First, although these patients were extracted from a large multicenter database, we cannot exclude risk of bias related to our matching approach and to differences in patient’s management across sites. Second, diabetic status was assessed based on previous medical history as reported by patients, relatives or consultants, so we did not have information on HBA1C levels or exhaustive diabetes complications. Whether having a poorly controlled diabetes is of worse prognosis or not will be the object of a further study. Third, we excluded patients with CKD. However, some of these patients may have been missed, as detailed information on pre-ICU renal function was not always available. In addition, some cases of prerenal AKI, or rapidly reversible AKI, may have been missed. Fourth, AKI was diagnosed according to the KGDIGO guidelines but only based on serum creatinine variations, as urinary output was not available in the database, which can not be corrected retrospectively [24]. Fifth, the use of RRT was not based on standardized criteria but was instead upon clinicians’ decision as standardized recommendations do not exist and as strategies differ across sites, like in most previous studies. Finally, no long-term follow-up was available in this database, which is a limitation as previous studies have shown that renal recovery must be evaluated no earlier than one year after an acute kidney injury episode [25].

No nephrologist consultant was involved in patients’ management. However, in all participating ICUs, at least one senior intensivist is a nephrologist. This point can be of interest as previous studies have shown that nephrologist follow-up improves all-cause mortality of severe acute kidney injury survivors [26].

Long-term follow-up including determination of diabetes control and analysis of urinary sediment is warranted.

In summary, in patients with severe sepsis or septic shock, diabetes is not associated with AKI or RRT but is an independent risk factor for failure to recover the previous level of renal function by ICU discharge. Involvement of a nephrologist in the early ICU care of patients with diabetes and severe sepsis or septic shock deserves consideration, as well as a long-term follow-up by a nephrologist after ICU discharge.
